# Predictors of favorable long-term outcomes in first-line surgery for microprolactinomas

**DOI:** 10.1007/s11060-025-04958-6

**Published:** 2025-02-04

**Authors:** Lukas Andereggen, Emanuel Christ

**Affiliations:** 1 Department of Neurosurgery, Kantonsspital Aarau, Aarau, 5001 Switzerland; 2 Facult of Medicine, University of Bern, Bern, Switzerland; 3 Department of Endocrinology, Diabetes and Metabolism, University Hospital of Basel, Basel, Switzerland

**Keywords:** Dopamine agonists, Long-term outcome, Microadenoma, Primary surgical therapy, Prolactinoma

## Abstract

**Purpose:**

Opting for first-line surgery in carefully selected patients with microprolactinomas provides the advantage of avoiding long-term dopamine agonist (DA) medication and potential associated side effects. However, the lack of comprehensive long-term data poses a challenge in identifying those patients who would benefit the most from upfront surgery. To improve guidance in the selection process for microprolactinoma patients in clinical practice, we aimed to establish simple clinical and biochemical parameters predicting non-dependence on DAs.

**Methodology:**

Retrospective analysis of a prospectively maintained database, focusing on patients with microprolactinomas who underwent upfront surgery. We assessed clinical and biochemical risk factors for the patients’ reliance on DAs at their latest follow-up using regression analysis. We next proceeded to conduct Receiver Operating Characteristic (ROC) analysis to determine the optimal threshold cutoff prolactin (PRL) level for practical application in clinical settings that best differentiates between surgical long-term remission status and long-term dependence on DAs.

**Results:**

A microadenoma was observed in 46 patients, of whom 12 (26%) exhibited long-term dependence on DAs at a median follow-up of 78 months. Baseline PRL values were significantly higher in patients with long-term DA dependence compared to those without (*p* = 0.05). High baseline PRL values (HR 23.9, 95% CI 1.0-593.7, *p* = 0.05), but not the presence of headache or male gender, were identified as independent predictors of long-term dependence on DAs. PRL thresholds for discriminating long-term DA dependence were estimated to be 290 µg/L (AUROC = 0.73, 95% CI 0.55–0.92, *p* = 0.03; sensitivity = 90%, specificity = 80%).

**Conclusions:**

In patients with microprolactinomas, first-line surgery presents a favorable prospect for reducing reliance on DAs. However, for those with high PRL levels ≥ 290 µg/L at diagnosis, first-line surgery is not recommended, as the majority of them require adjuvant DA therapy in the long term.

## Introduction

Prolactinomas present distinctive therapeutic difficulties for healthcare professionals due to the ongoing challenge of achieving long-term cure and accurately predicting rates of sustained remission [1, 2]. Dopamine agonists (DAs) effectively normalize prolactin (PRL) levels and reduce prolactinoma size in most cases, with resistance to cabergoline being rare [3]. However, the potential adverse effects associated with long-term DA therapy emphasize the need to explore alternative treatment approaches such as surgery [4–9]. Therefore, the current consensus statement by the Pituitary Society recommends surgery as the primary treatment for microprolactinomas and well-defined macroprolactinomas classified as Knosp grade 0 and 1 [10]. This represents a notable departure from previous recommendations, which favored treating prolactinomas with DAs as the first-line approach [11–14] and reserving surgery for cases of DA resistance or intolerance, cystic adenomas, or intratumoral hemorrhage causing persistent visual disturbances [15–21].

Despite this emerging trend, there is a lack of comprehensive long-term data, making it challenging to identify the ideal surgical candidates with microprolactinomas. This is crucial given the primary objective of achieving long-term independence from DAs, as medical therapy provides an effective and safe alternative. Therefore, for interdisciplinary case discussions and subsequent patient information, it is important to carefully select those patients best suited for primary surgery. Thus, our objective was to define the optimal patient cohort with microprolactinomas who would derive the greatest benefit from primary surgery, aiming to minimize the need for prolonged use of DAs postoperatively. To enhance guidance in this selection process in clinical practice, we aimed to establish simple clinical and biochemical parameters predicting non-dependence on DAs in the long-term.

## Patients and methods

### Study design

In this retrospective study, we analyzed data from prolactinoma patients recorded in our institution’s database, which had been maintained prospectively from January 1996 to December 2022. The dataset comprised all consecutive individuals who underwent upfront surgery for a microprolactinoma, excluding those who had undergone DA therapy prior to surgery. Approval for the project was obtained from the Human Research Ethics Committee of Bern (Kantonale Ethikkommission KEK Bern, Bern, Switzerland) under project numbers 10-10-2006 and 8-11-2006.

We conducted a comprehensive comparison, analyzing the demographic characteristics and surgical outcomes of two groups: those who achieved biochemical remission without long-term DA therapy and those who did not achieve remission and subsequently became dependent on DAs. For patients with elevated PRL levels (> 20 µg/L) and low gonadotropins at three months post surgery, DA therapy (e.g., bromocriptine, quinagolide, and primarily cabergoline) was initiated [22]. For patients with PRL levels only marginally above the normal range without impact on gonadal axis, DA therapy was not initiated, and PRL levels were monitored during routine follow-up. We assessed clinical factors such as age, gender, and symptoms at presentation, and biochemical risk factors (PRL values, endocrinopathies, tumor characteristics) for the patients’ reliance on DAs at their latest follow-up using regression analysis. We next proceeded to conduct Receiver Operating Characteristic (ROC) analysis to determine the optimal threshold cutoff PRL level for practical application in clinical settings that best differentiates between surgical long-term remission status and long-term dependence on DAs.

### Preoperative evaluation

#### Clinical examination

The diagnosis relied on preoperative clinical and biochemical evaluations, following a standardized protocol for pituitary magnetic resonance imaging (MRI) outlined below. Baseline characteristics encompassed patients’ age, gender, body mass index (BMI), and the clinical manifestations prompting their presentation (i.e., headache, visual deficits, galactorrhea, amenorrhea in women, low libido/erectile dysfunction in men) [23].

#### Biochemical evaluation

PRL levels were evaluated using the immunoradiometric PRL assay, employing serum dilution to overcome the high-dose PRL hook effect [24]. The presence of macroprolactin was regularly examined [25]. The upper limit for PRL levels was set at 20 µg/L [26]. In terms of pituitary axis deficits, partial hypopituitarism was defined as impaired secretion of one or more pituitary hormones. Secondary adrenal insufficiency was identified by low cortisol levels (< 50 nmol/L) in the serum or normal cortisol levels with inadequate responses to the adrenocorticotropin (ACTH) stimulation test or insulin tolerance test. The diagnosis of secondary hypothyroidism relied on finding low-normal thyroid-stimulating hormone (TSH) levels and a low free thyroxin (FT4) level. A deficiency in gonadotropins or central hypogonadism was considered if gonadotropin levels were low-normal alongside low estradiol/testosterone levels.

#### Neuroimaging assessment

MRI scans were conducted using a 1.5- or 3-Tesla system, encompassing a Proton/T2-weighted whole-brain examination that included unenhanced, contrast-enhanced, dynamic contrast-enhanced, and post-contrast-enhanced overlapping studies in the axial, sagittal, and coronal planes focused on the sellar region [27–30]. A tumor with a diameter ranging from 1 to 10 mm was categorized as a microadenoma. The invasiveness of the cavernous sinus was described using the Knosp classification [31, 32]. Immunohistochemical confirmation with a PRL antibody, in line with the WHO classification of neuroendocrine tumors, served as the gold standard for diagnosis [33].

### Indication for upfront surgery

During the weekly interdisciplinary pituitary tumor board meeting, all patients were discussed regarding the possibility of performing upfront surgery, aiming to avoid long-term reliance on DAs. The decision for immediate surgery was further discussed with patients, taking into account their preference for surgical intervention over prolonged DA therapy. In contrast to the other countries, where financial factors may impact surgical decisions for prolactinoma patients with lower income [34], Switzerland’s universal health coverage ensures that treatment choices, whether medical or surgical, are not influenced by financial considerations [35].

The pituitary surgery was performed using a primarily microsurgical transseptal, transsphenoidal approach, followed by sellar reconstruction [36]. Over time, this method was enhanced with the addition of a microsurgical endoscopic-assisted technique in a few patients. Postoperative complications (i.e. cerebrospinal fluid leaks, infections, cranial nerve deficits, epistaxis, Syndrome of Inappropriate Antidiuretic Hormone Secretion (SIADH), and the onset of diabetes insipidus (DI) were noted.

### Follow-up assessment

The initial follow-up occurred three months post-pituitary surgery. For patients exhibiting elevated PRL levels (> 20 µg/L), DAs was initiated [37]. In cases where PRL levels were slightly elevated but without clinical symptoms, DA therapy was not initiated, and PRL levels were monitored during routine follow-up. Last follow-up referred to the most recent documented visit to the endocrine outpatient clinic. DAs were gradually tapered 24 months after starting medical therapy if PRL levels had normalized [38, 39]. There was no standard follow-up to monitor tumor size through sellar MRI [40, 41]. Patients were considered to be in remission if their PRL levels were below 20 µg/L during follow-up.

### Statistical analyses

Data were analyzed using IBM SPSS statistical software (V29.0 Software, IBM Corp., New York, NY, USA), and GraphPad Prism (V9.0 software, San Diego, CA, USA). Continuous variables were assessed for homogeneity of variance and are presented as mean ± SD unless stated otherwise. Serum PRL levels are reported as median values with interquartile range (IQR, 25th to 75th percentile). Categorical variables are expressed as numbers and percentages. To compare means between groups, Student’s t-test was employed for normally distributed data, while the Mann–Whitney test was used for nonparametric data. The Wilcoxon signed-rank test was utilized to assess paired differences in PRL levels before and after treatment. Categorical variables were compared using Pearson’s chi-square test or Fisher’s exact test, as appropriate. The Spearman rank-order correlation coefficient was calculated to check for the strength of association between different variables (i.e., PRL, DA dependency). The analysis included evaluating the proportion of patients with long-term dependence on DAs and conducting time-dependent multivariable regression analysis to calculate hazard ratios (HR) for potential risk factors. The variables tested encompassed age at diagnosis, sex, headache at presentation, gonadotropin deficiency, baseline BMI (kg/m^2^), and initial PRL levels. In the multivariable regression analysis, all dependent risk factors in the univariable regression with a p-value ≤ 0.3 were included. Baseline PRL values were log-transformed before being imputed in the regression analysis due to the positively skewed distribution of the data. The significance level was set at 5%.

## Results

### Patient cohort at diagnosis

In total, 87 prolactinoma patients underwent upfront surgery without prior DA therapy during the study period. Forty-one patients with macroprolactinoma were excluded from the study. The remaining cohort consisted of forty-six patients meeting the inclusion criteria, comprising six men and forty women. Patient characteristics at the time of diagnosis in this cohort are outlined in Table 1.

**Table 1:** Patient characteristics at baseline

Baseline Patient Characteristics	Absence of DAs at last follow-up	Presence of DAs at last follow-Up	Total	*P* value
Number of patients, n (%)	34 (74)	12 (26)	46 (100)	
Age at diagnosis in years (mean ± SD)	33.1 ± 8.7	32.3 ± 5.6	32.9 ± 7.9	0.77
Women, n (%)	30 (88)	10 (83)	40 (87)	0.64
BMI (kg/m2 ± SD)	25.0 ± 5.9	26.2 ± 5.3	25.4 ± 5.7	0.65
Headache, n (%)	4 (12)	4 (36)	8 (18)	0.09
Affected pituitary axes, n (%)				
Gonadotropin deficiency	25 (74)	8 (67)	33 (72)	0.72
Secondary hypothyroidism	1 (3)	1 (8)	2 (4)	0.46
Secondary adrenal insufficiency	1 (3)	1 (9)	2 (5)	0.46
Cavernous sinus infiltration	0 (0)	1 (8)	1 (2)	0.26
Prolactin levels in µg/L (median; IQR)	108.9 (60–166)	199 (133–270)	133 (70–200)	**0.05**

BMI, body mass index; DA, dopamine agonists; n, numbers; SD, standard deviation; IQR, interquartile range

**Fig. 1:**
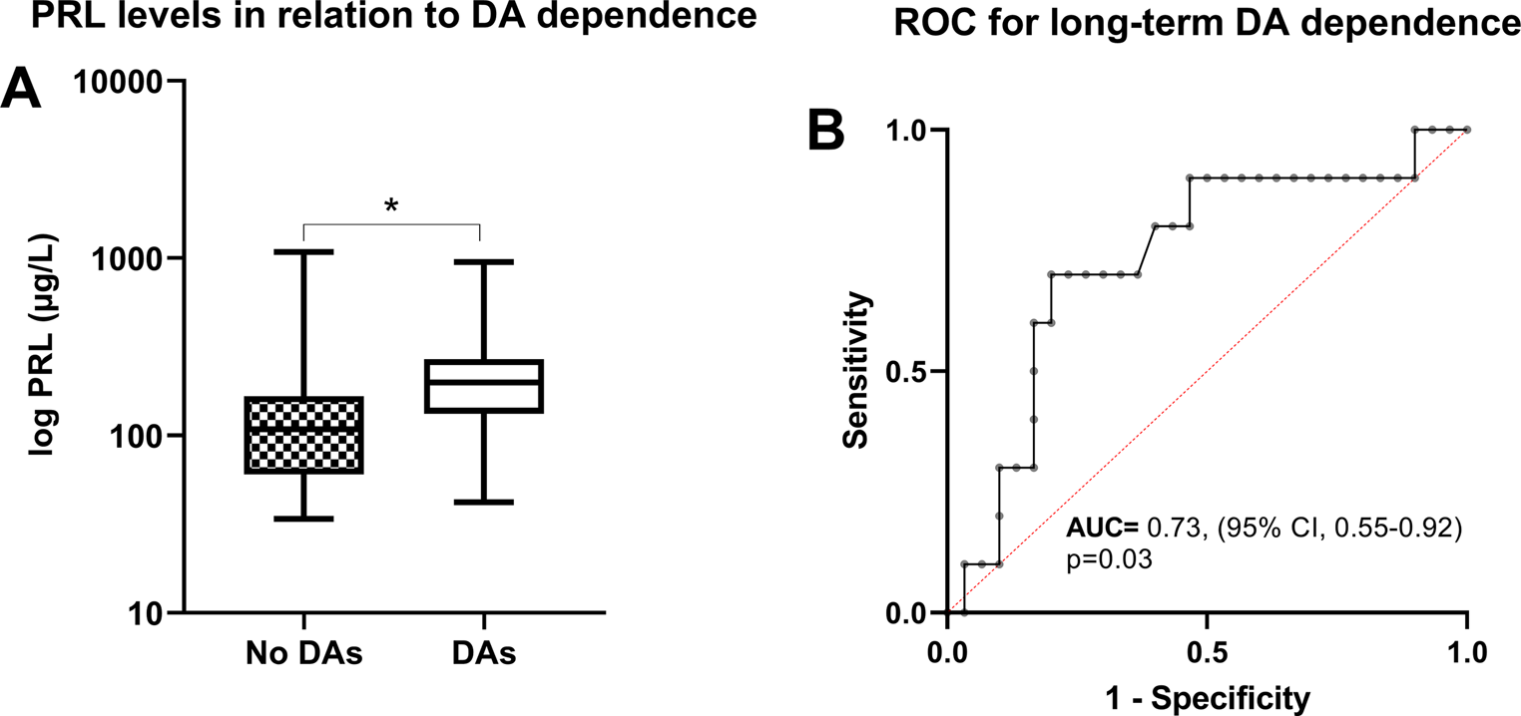
Prolactin levels for long-term dopamine agonist dependence in patients with microprolactinomas. (**A**) Patients without long-term dependence on DAs exhibited significantly lower baseline PRL levels compared to those with DA dependence (108.9 µg/L, IQR 60–166 µg/L vs. 199 µg/L, IQR 133–270 µg/L, *p* = 0.05). Receiver operating curves (ROC) for long-term dopamine agonist dependence in patients with microprolactinomas. (**B**) The estimated PRL threshold for discriminating long-term DA dependence was 290 µg/L (AUROC = 0.73, 95% CI 0.55–0.92, *p* = 0.03; sensitivity = 90%, specificity = 80%)

Frequent symptoms reported were headaches in 18% of cases, amenorrhea or irregular menstrual cycles in 81% of female patients, galactorrhea in 61% of cases, and a reduction in libido or erectile dysfunction in 50% of male patients. The mean (± SD) age of our cohort was 32.9 ± 7.9 years. None of the microprolactinomas exhibited signs of cavernous sinus invasion. Patients without long-term dependence on DAs showed a non-significant trend toward lower BMI and a reduced incidence of headaches compared to their counterparts with long-term DA dependence. Additionally, this group displayed significantly lower baseline PRL levels than those with DA dependence (Fig. 1A). No significant differences in the prevalence of gonadotrophic, thyrotrophic, and corticotrophic insufficiency were observed between the two cohorts.

There were no deaths among patients with a microadenoma. Surgical morbidity included rhinoliquorrhea in one patient, requiring transsphenoidal revision with autologous fat graft and dural patching. Two patients experienced transient hyponatremia. Post-surgery, one patient (2%) developed thyrotrophic insufficiency, and another (2%) developed corticotrophic insufficiency, contrasting with their baseline conditions. There were no reported instances of vascular injuries, meningitis, or abscesses.

### Early and long-term follow-up

Early follow-up took place three months after pituitary surgery. Thereby, serum PRL values decreased significantly, from 133 µg/L (IQR 70–199 µg/L) to 13 µg/L (IQR 6–34 µg/L), *p* < 0.001. Postoperative normalization of PRL levels was obtained in 32 patients (74%). None of the patients were lost to long-term follow-up. After a median follow-up of 78 months (IQR 31–147 months), there was a significant decrease in PRL values along with the control of hypogonadism. No significant differences in other endocrinopathies (i.e., secondary hypothyroidism, secondary adrenal insufficiency) were observed. Remission achieved withupfront surgery alone (without the need for long-term DA therapy) was observed in 31 patients (71%), while recurrence was noted in 13 patients (29%). DA therapy was ultimately required in 12 (26%) patients, with 3 (7%) receiving bromocriptine and 11 (19%) receiving cabergoline. Moreover, the prevalence of headaches significantly improved following the control of hyperprolactinemia in the long term (Table 2).

**Table 2:** Patient characteristics at last follow-up

Patient characteristics	Baseline	Last follow-up	*P* value
BMI (kg/m2 ± SD)	25.4 ± 5.7	24.7 ± 5.7	0.54
Headache, n (%)	8 (18)	1 (2)	**0.01**
Gonadotropin deficiency, n (%)	33 (72)	5 (18)	**< 0.001**
Prolactin levels in µg/L (median; IQR)	133 (70–200)	12.2 (7.3–21.3)	**< 0.001**

BMI, body mass index; n, numbers; SD, standard deviation; IQR, interquartile range

We next sought to analyze the correlation between serum PRL levels and DA dependency in the long term. The Spearman rank-order correlation coefficient revealed a significant positive correlation between all patients’ PRL and DA dependency (*r* = 0.4, *p* = 0.03).

We subsequently conducted a regression analysis (Table 3) to assess clinical and biochemical risk factors contributing to patients’ reliance on DAs during their latest follow-up.

**Table 3:** Predictors of long-term dependence on dopamine agonists

Predictors	Univariable analyses HR (95% CI)		*P* value		Multivariable analyses HR (95% CI)		*P* value	
Age (years)	1.0 (0.9–1.1)	0.92						
Sex (female)	3.3 (0.7–16.6)	0.15		1.2 (0.1–16.4)		0.9		
Headache (baseline)	5.4 (1.4–20.3)	0.01		2.7 (0.6–13.5)		0.22		
Gonadotropin deficiency, n (%)	3.2 (0.8–12.9)	0.11		3.3 (0.4–26.9)		0.27		
Baseline BMI (kg/m2)	1.0 (0.9–1.2)	0.64						
PRL levels (baseline)	38 (2.4–603.0)	0.01		23.9 (1.0-593.7)		**0.05**		

BMI, body mass index; CI, confidence intervals; DA, dopamine agonist; HR, hazard ratio; PRL, prolactin

Univariable analyses revealed that the presence of headaches (HR 5.4, 95% CI 1.4–20.3, *p* = 0.01), rather than baseline BMI levels or patient age, significantly predicted persistent DA dependence in the long term. Furthermore, multivariable analyses demonstrated that high baseline PRL values (HR 23.9, 95% CI 1.0-593.7, *p* = 0.05), rather than the prevalence of headaches at diagnosis or gonadotropin deficiency, were independent risk factors for persistent DA dependence in the long term. Given these results, with baseline PRL levels being an independent risk factor for persistent DA dependence in microprolactinoma patients, we then proceeded to conduct ROC analysis. This aimed to determine the optimal threshold cutoff PRL level for practical application in clinical settings, effectively distinguishing between surgical long-term remission status and long-term dependence on DAs. Thus, it optimizes the prediction of potential non-remission in patients with prolactinoma who underwent surgery but remain dependent on long-term DA therapy. Thereby, PRL thresholds for discriminating long-term DA dependence were estimated to be 290 µg/L (AUROC = 0.73, 95% CI 0.55–0.92, *p* = 0.03; sensitivity = 90%, specificity = 80%, Fig. 1B).

## Discussion

The current analysis, employing a surgery-first approach in microprolactinomas, indicates that, over the long term with a median follow-up of 7 years:

i) Upfront surgery resulted in a high likelihood of avoiding long-term DA therapy.
ii) Only baseline PRL levels were independently associated with persistent DA reliance.
iii) Patients with microprolactinomas who have PRL values of > 290 µg/L are likely to require long-term supplemental DA therapy.

In prolactinomas, healthcare professionals face challenges regarding the difficulty of achieving long-term cures and accurately predicting sustained remission rates. In recent years, there has been an increasing preference for TSS as a first-line approach in selected prolactinoma patients due to its favorable outcomes and the documented adverse effects associated with long-term DA therapy [5, 42–45]. This approach gained support from recent meta-analyses confirming successful disease remission through surgery in the majority of patients [17, 18], and led to the initiation of the (NCT 04107480) to investigate whether TSS for prolactinoma resection is superior to standard care as either a first-line or second-line treatment [22]. Similarly, the most recent Consensus Statement by the Pituitary Society has shifted its attitude in the same direction, indicating a significant departure from past guidelines by discussing the possibility of surgery as the primary intervention, led by an expert surgeon, alongside DA therapy., especially for patients with microprolactinomas and well-defined macroprolactinomas [10]. However, the main challenge lies in the absence of comprehensive long-term data, which complicates the identification of ideal surgical candidates. Careful patient selection for upfront surgery becomes crucial, and defining the optimal patient cohort for first-line surgery is necessary to decrease reliance on DAs. This is particularly true for microprolactinomas, as they are considered to have the greatest potential among prolactinomas to avoid long-term DA therapy [6]. Therefore, during interdisciplinary discussions aimed at achieving consensus on the optimal therapy with regard to long-term outcomes, when first-line surgery is preferred and consented to, it is essential to establish clinical and biochemical parameters that predict long-term DA independence. Thus, we included parameters potentially indicative of long-term dependence on DAs, such as age, gender, BMI, presence of hypogonadism, and headaches. Specifically, elderly patients with prolactinoma exhibited notably higher PRL levels compared to adolescents [46–48]. Additionally, we observed a significant positive correlation between patients’ PRL values and their age or BMI [46]. There exists a complex relationship between hyperprolactinemia and metabolic syndrome in prolactinoma patients, with the normalization of PRL levels associated with a reduction in BMI [49]. Prolactinoma patients often have elevated BMI, but normalizing PRL levels through DAs or surgery, or both is associated with a reduction in body weight, suggesting a potential link between PRL regulation and metabolic improvements [49–51]. In terms of gender, men with prolactinomas frequently experience prolonged hyperprolactinemia compared to women, who often manifest more obvious symptoms like amenorrhea, promptly investigated [52]. Importantly, our univariable analysis indicates that the presence of headaches, rather than baseline BMI levels or patient sex, significantly predicts persistent DA dependence in the long term. This finding is intriguing, as headaches in adenoma patients may be influenced by various factors including family history and hormonal activity of the adenoma [53]. In prolactinomas, however, headaches are not uncommon [54], and studies link them to high PRL levels and migraine [55]. Thereby, DAs show promise in treating these headaches, with evidence suggesting a role of hyperprolactinemia in their pathogenesis [56]. Interestingly, there is a reported relationship between high PRL levels and migraine attacks in patients with microprolactinoma [57], indicating that the adenoma entity, rather than its size, may be responsible for the effect, and potentially suggesting that long-term dependence on DAs may serve as an indicator of headache persistence. Finally, our results of the multivariable analysis indicated that rather than the prevalence of headaches at diagnosis or gonadotropin deficiency, only high baseline PRL values were independent risk factors for persistent DA dependence in the long term. This is plausible even in patients with microprolactinomas, as those with high baseline PRL levels were associated with a poor treatment response to DAs in the long term [58]. Similarly, it has been suggested that achieving a low nadir PRL level post-surgery alongside extended treatment duration, may allow for the possibility of discontinuing DAs in the long term [59], corroborating our results with high initial remission rates. In addition, surgical techniques have vastly improved over the past few decades, and extracapsular dissection has added established benefits to the surgical armamentarium, including high remission rates, low cerebrospinal fluid leakage, and minimal endocrine deficits [60]. This method serves as a strong first-line alternative to DAs, particularly for patients with microprolactinomas of low Knosp grade [61]. Moreover, the endoscopic approach enables precise pseudocapsule identification, allowing for effective extracapsular resection, which offers superior outcomes compared to intracapsular resection in terms of gross total resection, biochemical remission, and recurrence rates, without increasing postoperative complications [62].

As high baseline PRL levels themselves were identified as an independent risk factors for persistent DA therapy, we proceeded to conduct ROC analysis to determine the optimal threshold cutoff PRL level for practical application in clinical settings, aiming to differentiate most effectively between surgical long-term remission status and long-term reliance on DAs. Thereby, patient derived the most substantial benefits from an initial surgical approach and exhibited the highest cure rate when their preoperative PRL values were < 290 µg/L. The reason why higher PRL levels pose a risk factor for surgical failure in small prolactinomas remains unknown. A previous study by Tyrell et al. also demonstrated a correlation between higher preoperative PRL levels and a lower likelihood of successful surgical outcomes, with only 37% of patients with PRL levels above 200 µg/L achieving success [63]. Similarly, a recent study by Osorio et al. identified a strong correlation between the volume of prolactinomas and serum PRL levels [2]. Individuals who did not achieve remission exhibited a more pronounced elevation in serum PRL for each increment in preoperative tumor volume, suggesting the presence of distinctive tumor characteristics [2]. Furthermore, remission rates were observed to be higher for midline-located prolactinomas compared to those positioned laterally [7]. Correspondingly, preoperative PRL levels below 200 µg/L were associated with a higher probability of achieving long-term remission [7]. Conversely, preoperative PRL levels exceeding 500 µg/L are improbable to result in surgical cure, and levels surpassing 1000 µg/L are unlikely to achieve biochemical control [64, 65].

It’s nonetheless important to stress that the primary goal is not to favor TSS over DAs, but rather to identify the most suitable surgical candidates with microprolactinoma. When considering first-line surgery, emphasis should also be placed on minimizing the complication rate, with preference given to experienced pituitary surgeons at high-volume centers. Additionally, considering their ease of administration, monitoring, and favorable tolerance, DAs provide a viable alternative [66]. Despite being low and in line with earlier investigations [67], our study reveals that morbidity, mortality, and the occurrence of new endocrinopathies are not completely absent. Nevertheless, it is advisable to exercise caution when prescribing DA without interdisciplinary agreement due to potential adverse effects [44, 45, 68, 69], including significant psychosocial consequences like financial burden and alterations in personality, which must be taken into account [44, 45]. Concerns about the long-term safety have surfaced regarding the cumulative use of DAs [69–71], with instances reported of hyperprolactinemia recurring after discontinuation [72–74]. Additionally, choosing a surgical-first approach without prior DA prescription may prove advantageous due to the potential for adenoma fibrosis associated with DA therapy [75, 76]. Although studies suggest that fibrosis may have a negative impact on surgical outcomes, the findings remain controversial [15].

In this regard, our rationale for excluding well-circumscribed macroprolactinomas—which are known to respond favorably to upfront surgery when not invading the medial wall of the cavernous sinus—was based on the consideration that these larger tumors often present distinct clinical challenges [42, 68]. Therefore, this study identified the long-term predictors in a cohort where long-term remission with upfront surgery by an expert pituitary neurosurgeon is anticipated. Surgery, considered alongside dopamine agonist treatment as a first-line option, can help surgeons make better decisions for achieving long-term remission in this specific subgroup of prolactinoma patients with microadenomas.

In summary, prolactinomas necessitate treatment for hyperprolactinemia to prevent complications like infertility, gonadal dysfunction, and osteoporosis [77]. This study indicates that microprolactinomas treated with upfront surgery show long-term remission rates with minimal morbidity in the majority of patients. Particularly during interdisciplinary discussions, when first-line surgery is preferred and consented to in order to avoid long-term DA therapy in patients with a microadenoma, a threshold below 290 µg/L can be considered a robust indicator of surgical success in patient education.

## Study limitations

Due to the extended follow-up duration, data on pituitary insufficiency are lacking for certain individuals. Additionally, the outcomes of long-term dependence on DAs may have been influenced by a follow-up period of less than 24 months in a subset of patients. Our treatment approach involved gradually tapering medications after 24 months of initiating medical therapy, provided that PRL levels had normalized or an adenoma reduction of over 50% was achieved. However, a notable strength of our study lies in the remarkable homogeneity of our series, spanning 25 years, with consistent indications, treatment modalities, and follow-up protocols. Histopathological examinations were performed on all surgical patients starting in 1996. However, individual results were not systematically documented in the registry, which limited our ability to retrospectively confirm histopathologically diagnosed prolactinomas for all 46 patients. To minimize the potential impact of stalk effects in our study, we established stringent diagnostic criteria, including serum prolactin levels above 200 µg/L and MRI findings characteristic of prolactinomas. Furthermore, we meticulously reviewed MRI images to rule out the possibility of stalk mass effects in cases with borderline prolactin levels. Another limitation of our study is the absence of data on the Ki-67 index and granulation patterns in the prolactinomas analyzed. These parameters may provide insights into tumor aggressiveness and clinical outcomes [10, 77–79]. Future studies that incorporate these markers will be crucial in further elucidating the biological behavior and long-term prognostic implications of prolactinoma treatment.

Although we acknowledge the potential limitations of initially relying on a primarily microsurgical approach, supplemented over time by a microsurgical endoscopic-assisted technique in a few patients in this historical patient cohort, it is essential to emphasize the remarkable advancements in high-definition surgical endoscopes and highly reliable neuronavigation systems that have since transformed these methods [80]. These innovations have broadened surgical possibilities, enabling more complete tumor resections with reduced complication rates [81–85]. The debate continues regarding the expansion of first-line surgical indications for prolactin-producing tumors, particularly in patients with microprolactinomas, which offer a promising opportunity to reduce reliance on DAs [42, 86].

## Conclusion

In patients with microprolactinomas, first-line surgery offers a promising opportunity to decrease dependence on DAs. However, for patients with elevated PRL levels ≥ 290 µg/L at diagnosis, upfront surgery cannot be recommended, as the majority of them necessitate long-term adjuvant DA therapy. This clarification is essential for establishing a profile to determine whether these patients should directly follow the established approach, which advocates DAs as the first-line treatment, or if upfront surgery offers a more beneficial long-term outcome.

## Data Availability

The authors agree to share data upon reasonable request.

## Abbreviations

DA: Dopamine agonist
MRI: Magnetic resonance imaging
PRL: Prolactin
TSS: Transsphenoidal surgery
